# Modified peroral endoscopic myotomy for a bilobed Zenker’s diverticulum: one incision, three tunnels, and two myotomies

**DOI:** 10.1055/a-2960-2272

**Published:** 2026-10-02

**Authors:** Pieter Jan Poortmans, Andrea Sorge, Maria Eva Argenziano, Lobke Desomer, David J. Tate

**Affiliations:** 1 Department of Gastroenterology and Hepatology 60200 University Hospital GhentGhentFlandersBelgium; 2 Department of Internal Medicine and Pediatrics 26656 Ghent UniversityGhentFlandersBelgium; 3 Gastroenterology and HepatologyUniversity Hospital BrusselsBrusselsBelgium; 4 Department of Surgery, Oncology and Gastroenterology 9308 University of PaduaPaduaItaly; 5 Gastroenterology Unit 18624 Padua University HospitalPaduaItaly; 6 Department of Gastroenterology and Hepatology 192827 AZ Delta vzwRoeselareFlandersBelgium

Zenker’s diverticulum (ZD) is a pulsion pseudodiverticulum arising at Killian’s triangle, driven by impaired relaxation and compliance of the upper esophageal sphincter (UES). 1​ Peroral endoscopic myotomy for ZD (Z-POEM) uses a submucosal tunnel to expose and completely divide the cricopharyngeal septum and has become a preferred minimally invasive treatment, with technical and clinical success exceeding 90%. 2​ ​3​ We present a case of a bilobed ZD, a rare variant with two pouches divided by a septum treated by a modified Z-POEM approach ( Video 1 ).

**Video 1** A modified Z-POEM approach for a bilobed Zenker’s diverticulum: single-incision, three-tunnel access with sequential myotomies of the interlobular septum and the upper esophageal sphincter, and clip closure. Video URI:


A 75-year-old man presented with 6 months of progressive solid-food dysphagia and 10 to 12 kg of weight loss (Kothari-Haber score 6). Barium swallow and endoscopy confirmed a bilobed ZD above the UES, with a muscular septum between the lobules attached to the UES ( Fig. 1 ).

**Fig. 1:**
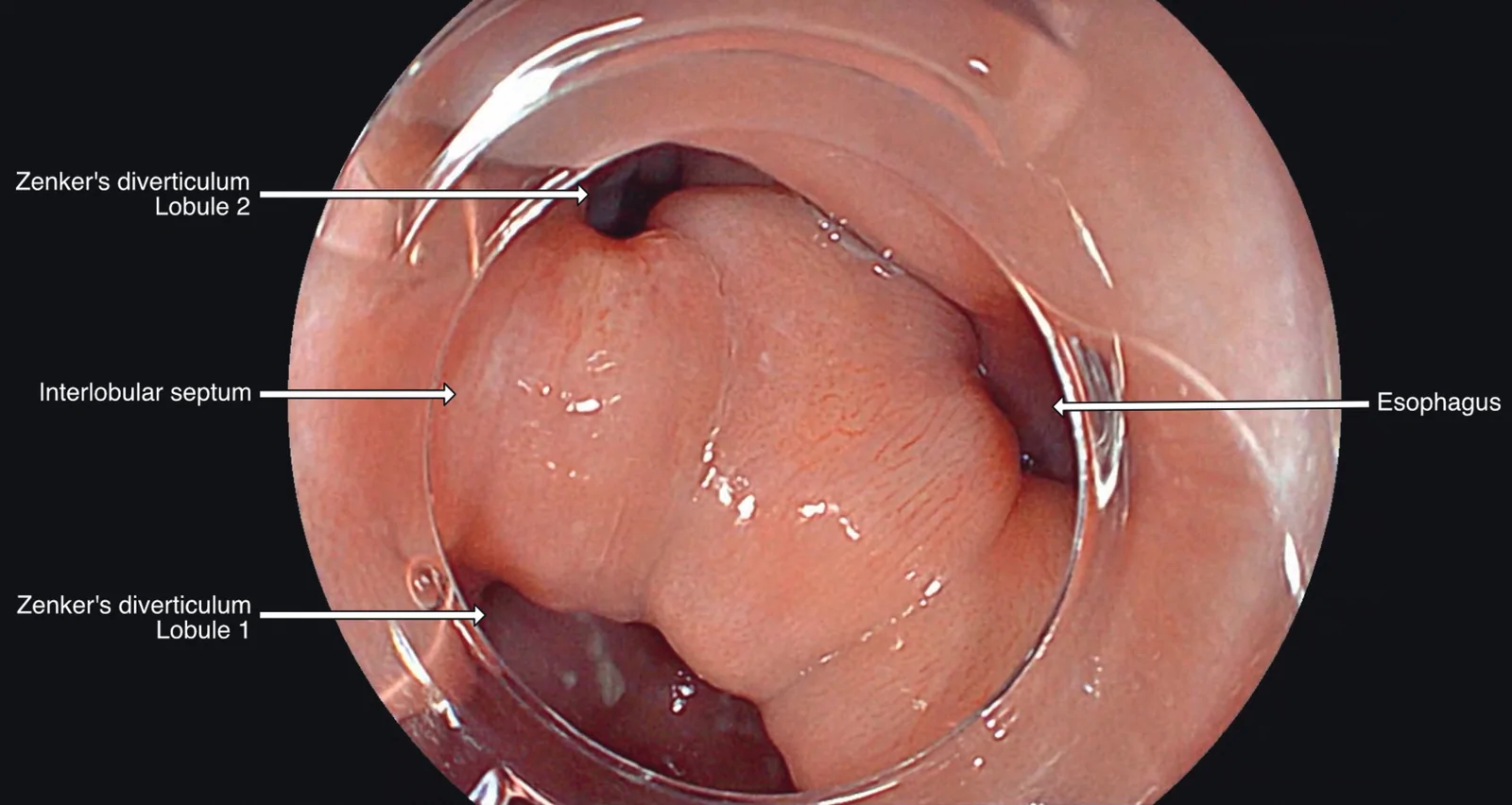
A bilobed Zenker’s diverticulum on endoscopy: two distinct pouches above the upper esophageal sphincter, separated by a muscular interlobular septum.

Z-POEM was performed under general anesthesia ( Fig. 2 ). After clearing retained food, a single longitudinal mucosal incision was made over the UES at the connection of the interlobular septum, through which three submucosal tunnels were created with a TriangleTip-J knife (Olympus, Japan): one within each diverticular lobule and a third along the esophageal side of the UES ( Fig. 3 ). Myotomy was then performed in two stages: first the interlobular septum and then the UES, with a 2-cm extension into the esophageal muscle beyond the cricopharyngeus ( Figs. 4 5 ). Hemostasis was achieved with soft coagulation, and the tunnels were closed with eight clips. The procedure time was 44 minutes.

**Fig. 2:**
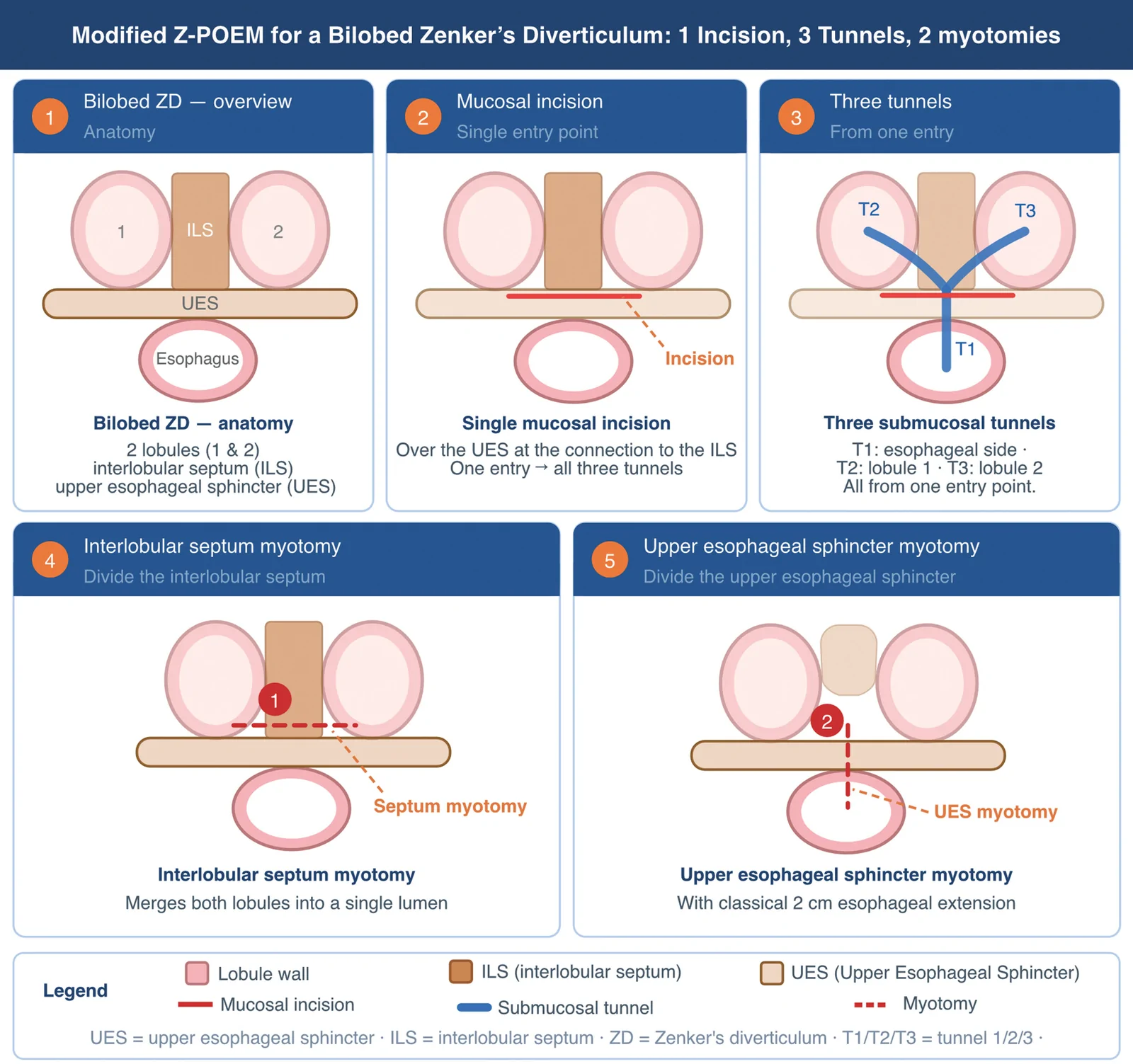
Schematic of the modified Z-POEM technique: single mucosal incision, three submucosal tunnels, and two-stage myotomy (interlobular septum and then the typical septum with 2 cm esophageal extension). ILS, interlobular septum; UES, upper esophageal sphincter; ZD, Zenker’s diverticulum.

**Fig. 3:**
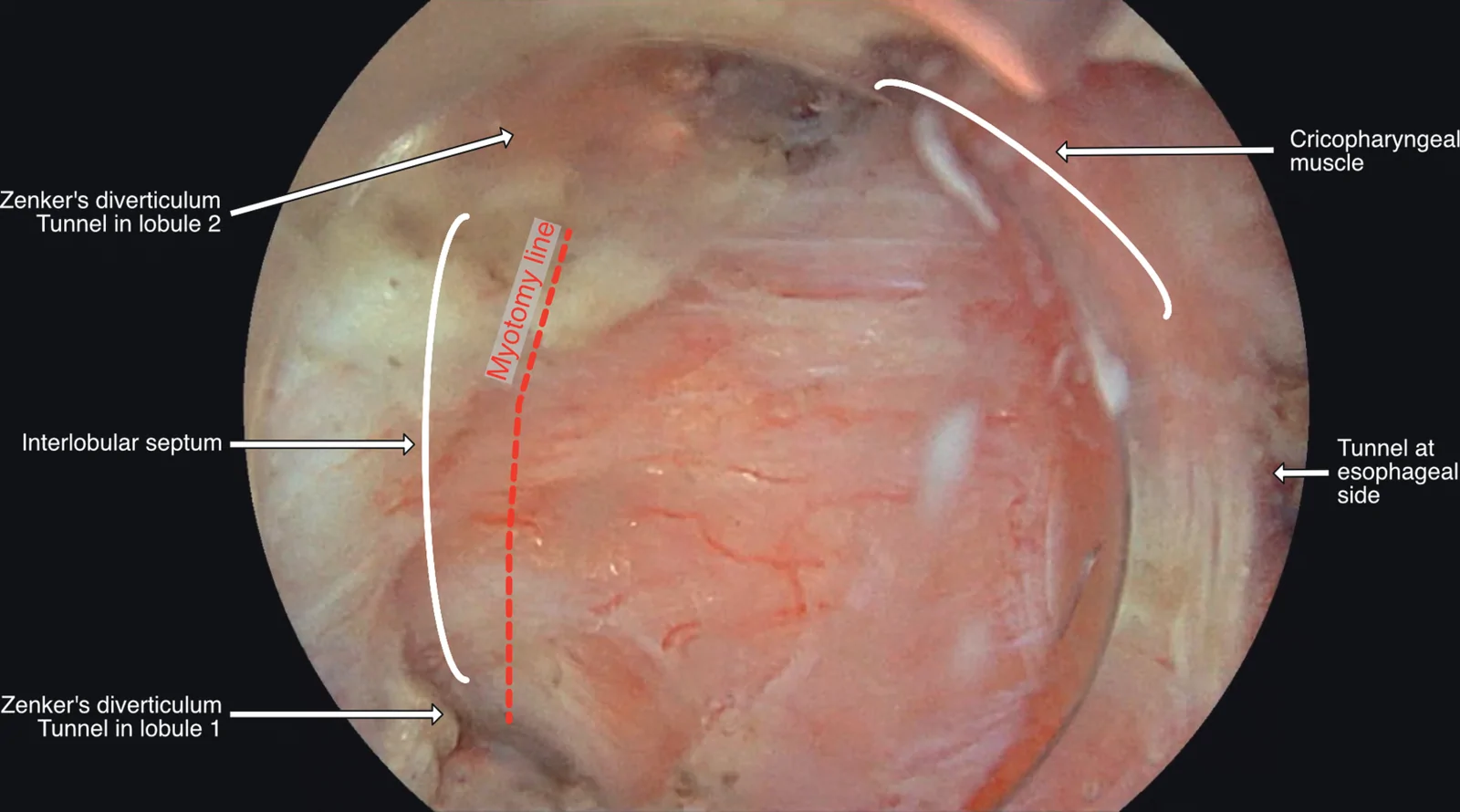
An endoscopic view of muscular bridges encountered during the peroral endoscopic myotomy procedure for a bilobed Zenker diverticulum with the interlobular septum and cricopharyngeal muscle exposed and isolated by tunnelling on all sides of the muscular brides. The line where the first myotomy will be performed is marked as a dashed red line.

**Fig. 4:**
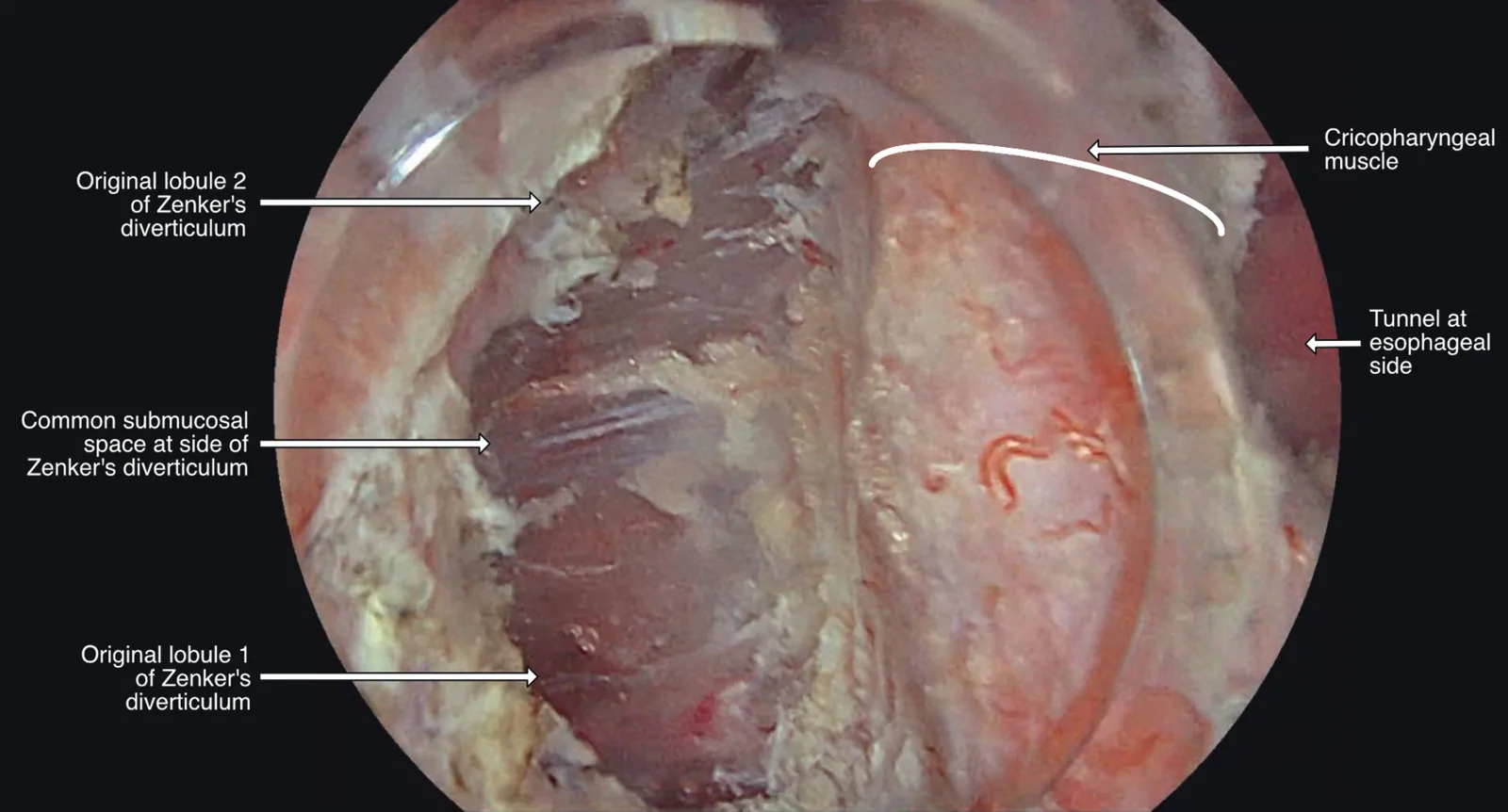
An endoscopic view of the submucosal space at the diverticular side after the myotomy of the interlobular septum during the peroral endoscopic myotomy procedure for the bilobed Zenker diverticulum.

**Fig. 5:**
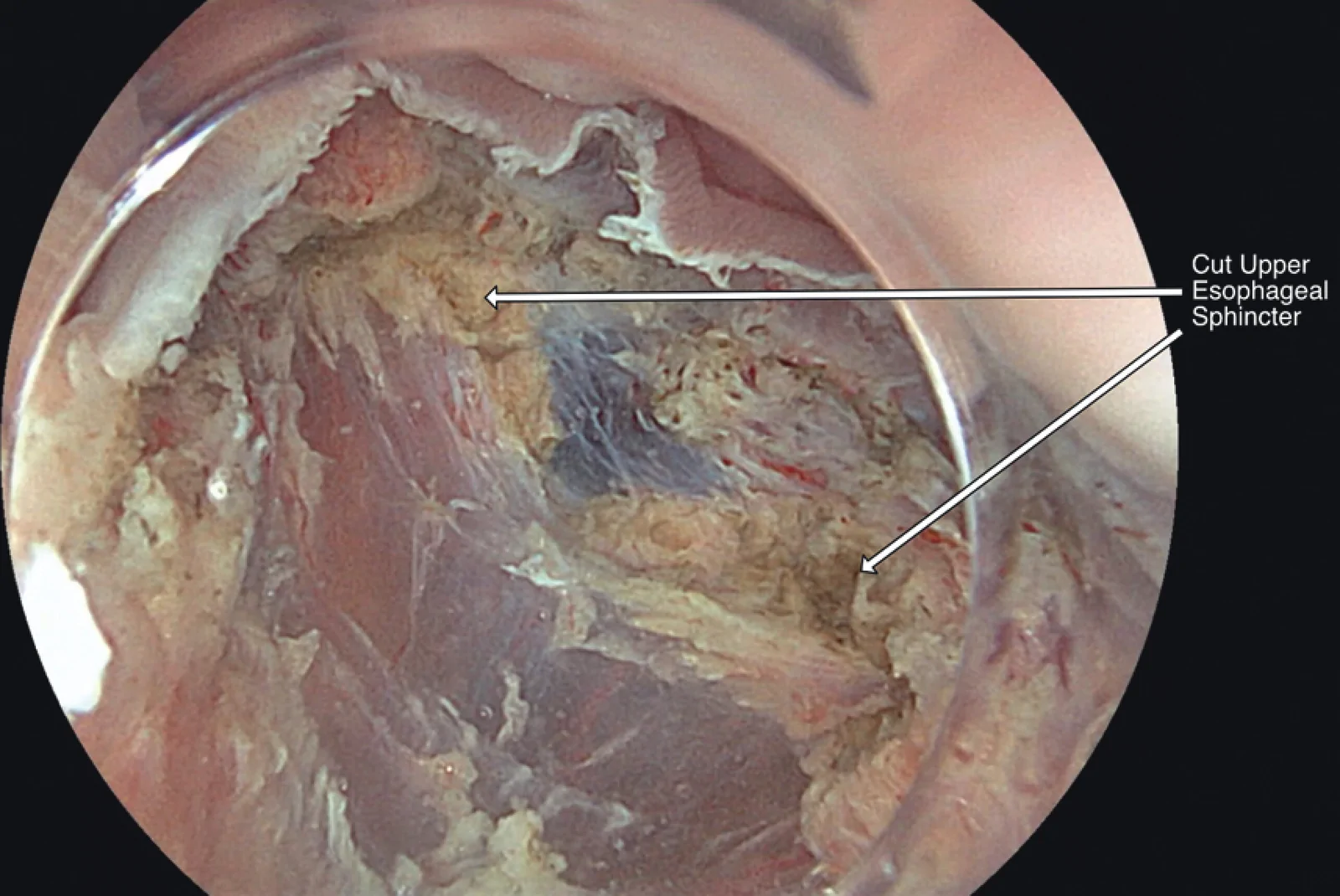
An endoscopic view of the end of the myotomy of the upper esophageal sphincter during the peroral endoscopic myotomy procedure for the bilobed Zenker diverticulum.

The procedure was uneventful. At post-procedure follow-up, the patient was symptom-free, with the Kothari-Haber score improving from 6 to 2. To our knowledge, this is the first reported Z-POEM for a bilobed ZD, demonstrating that the technique can be adapted to complex diverticular anatomy via a single mucosal entry point, multiple targeted tunnels, and sequential septal myotomy.

Endoscopy_UCTN_Code_TTT_1AO_2AD

## Declaration of GenAI Use

The authors confirm that no generative artificial intelligence was used in the preparation of this manuscript.
